# The effect of a specialized dyslexia font, OpenDyslexic, on reading rate and accuracy

**DOI:** 10.1007/s11881-016-0127-1

**Published:** 2016-03-18

**Authors:** Jessica J. Wery, Jennifer A. Diliberto

**Affiliations:** 1 School of Education, Elon University, 2105 Campus Box, Elon, NC 27244 USA; 2 School of Education, University of North Carolina at Chapel Hill, CB#3500, 201F Peabody, Chapel Hill, NC 27599-3500 USA

**Keywords:** Decoding, Dyslexia, Fluency, Font, Learning disabilities, OpenDyslexic, Reading

## Abstract

A single-subject alternating treatment design was used to investigate the extent to which a specialized dyslexia font, OpenDyslexic, impacted reading rate or accuracy compared to two commonly used fonts when used with elementary students identified as having dyslexia. OpenDyslexic was compared to Arial and Times New Roman in three reading tasks: (a) letter naming, (b) word reading, and (c) nonsense word reading. Data were analyzed through visual analysis and improvement rate difference, a nonparametric measure of nonoverlap for comparing treatments. Results from this alternating treatment experiment show no improvement in reading rate or accuracy for individual students with dyslexia, as well as the group as a whole. While some students commented that the font was “new” or “different”, none of the participants reported preferring to read material presented in that font. These results indicate there may be no benefit for translating print materials to this font.

## Introduction

An estimated 15–20 % of English-speaking school-aged children experience difficulty learning to read (International Dyslexia Association (IDA), 2007; Lyon, Shaywitz, & Shaywitz, 2003). Within the USA, the prevalence rate of dyslexia is estimated to be between 10 and 15 % (Fletcher, Lyon, Fuchs, & Barnes, 2007; Eden & Moats, 2002). Since its discovery, dyslexia has been highly researched and debated (Washburn, Binks‐Cantrell, & Joshi, 2013). Researchers in a variety of disciplines, including medicine, psychology, and education, have contributed to our understanding of dyslexia, as well as the methods and interventions that are effective for students with this disability.

Several authorities have put forth definitions of dyslexia. IDA has adopted the definition of dyslexia published by Lyon et al. (2003):

Dyslexia is a specific learning disability that is neurological in origin. It is characterized by difficulties with accurate and/or fluent word recognition and by poor spelling and decoding abilities. These difficulties typically result from a deficit in the phonological component of language that is often unexpected in relation to other cognitive abilities and the provision of effective classroom instruction. Secondary consequences may include problems in reading comprehension and reduced reading experience that can impede growth of vocabulary and background knowledge (p.2).

Dyslexia is also included within the Individuals with Disabilities Education Act (IDEA, 2004), within “specific learning disability (SLD)”, one of the 13 disability categories. The Diagnostic and Statistical Manual of Mental Disorders (DSM-V; American Psychiatric Association, 2013) includes dyslexia within “learning disorder.” Unfortunately, the inconsistency in terminology and the lack of one universally agreed upon definition of dyslexia has caused some confusion among special educators, administrators, and parents. However, most agree that dyslexia is a distinct type of SLD that presents in a difficulty with phonological coding (Shaywitz et al., 2004; Snowling, 2009).

As reading and writing have become increasingly crucial for success in and out of school, students with dyslexia are often at-risk for academic failure, lower reading self-efficacy (Burden, 2008), and lower self-esteem (Alexander-Passe, 2006) as well as an increased risk of dropping out of school (U.S. Department of Education, Office of Special Education and Rehabilitative Services, Office of Special Education Programs, 2006). Due to the potential for poor school and post-school outcomes, teachers, parents, and advocates often feel desperate to locate and employ accommodations and interventions to help students with dyslexia read.

**Fig. 1:**
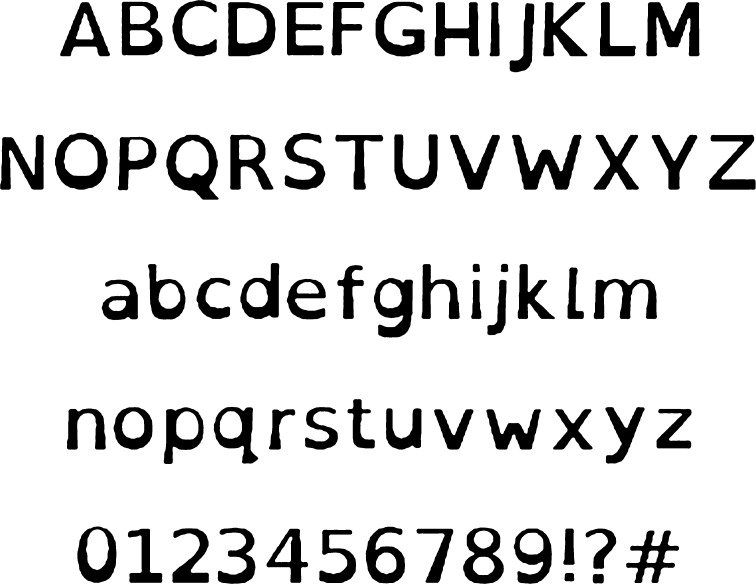
OpenDyslexic font. Source: BBC.com

Recently, two specialized typefaces or fonts “OpenDyslexic” (OD; Gonzalez, 2012) and “Dyslexie” (Boer, 2008) have been developed that purport to increase readability for those with dyslexia. These fonts differ from the other, more traditional fonts because the letters have been designed to have thicker or “heavier” lines near the bottom of the letters (See Fig. 1). The typeface developers of these fonts claim that this “heaviness” prevents the letters from turning upside down for readers with dyslexia, and makes it easier for people with dyslexia to distinguish individual letters while reducing reading errors and the effort it takes to read text (http://www.studiostudio.nl/en/information/).

Perhaps, the typeface developers developed this font based on the same misconception that dyslexia is characterized by letter reversals, what is commonly held by teachers (Washburn et al., 2013). However, four decades of research on dyslexia suggests reading difficulties stem from more basic deficits in alphabetic and phonological coding are the probable causes of the disorder rather than visual, semantic, or syntactic deficits (Vellutino, Fletcher, Snowling, & Scanlon, 2004).

Despite this erroneous foundation, these newly designed fonts have captured public attention across the world. National Public Radio, Scientific American, and eschool news (http://www.eschoolnews.com/2015/09/04/dyslexia-online-font-593/comment-page-1/#comment-230792) have featured interviews with one of the developers, and several websites contain numerous testimonials touting the benefits of the specialized fonts. Additionally, many of these websites highlight these testimonials on social media sites such as Facebook, heightening its visibility.

Today, the U.S. Department of Education places an increased emphasis on greater rigor in educational research (Institute of Educational Sciences, 2010), and legislative mandates call for the use of research-based practices in schools (NCLB, 2001; ESRA, 2002; IDEIA, 2004). These legal and policy changes as well as calls from the research academy (e.g., Horner et al., 2005; Gersten et al., 2005) further movements for evidence-based interventions, practices, or treatments in the field of special education (Odom, 2009; Odom et al., 2005), thus highlighting the importance of investigations into the efficacy of specialized fonts. Yet, no empirical research could be identified that investigated the effectiveness of the font with English readers, and very little research exists on its effectiveness in readers of other languages.

### Extant research

Two studies have investigated the effect of specialized fonts used with students with dyslexia. Rello and Baeza-Yates (2013) measured eye-tracking recordings of Spanish readers with dyslexia (aged 11–50) and found that OD did not significantly improve reading time nor shorten eye fixation. In her master’s thesis, de Leeuw (2010) compared Arial and Dyslexie with 21 Dutch students with dyslexia and found Dyslexie did not lead to faster reading, but may help with some dyslexic-related errors. To date, no peer-reviewed research studies report the use of a “dyslexia-friendly” font with English-speaking and English-reading students. Thus, research is needed to determine the effectiveness of a dyslexia-friendly font on reading rate and accuracy in English-speaking students identified with dyslexia prior to its widespread use.

Therefore, the purpose of the current investigation is to determine the effectiveness of a specialized dyslexia font on reading speed and accuracy on three reading tasks: (a) letter naming, (b) word reading, and (c) nonsense word reading. These three types of reading tasks were selected based on their strong correlation to reading achievement and ability to measure both reading accuracy and speed. The independent variables compared were the fonts (a) Arial, a san serif font; (b) Times New Roman (TNR), a serif font; and (c) OD, a specialized dyslexia font. Here, Arial and TNR both commonly used fonts are baseline conditions 1 and 2, respectively. OD is the treatment condition.

## Method

The current investigation utilized a single-subject alternating treatment design to determine the differences among three separate fonts within three reading tasks in elementary school students identified with dyslexia. Single-subject research is considered a rigorous, scientific experimental methodology used to establish evidence-based practices. The purpose of single-subject designs is to document functional relationships between independent and dependent variables thus focusing on practical significance as an outcome (Horner et al., 2005). Single-subject research data is typically compared systematically thought visual representations within and across conditions of a study using graphic representations (Alberto & Troutman, 2009).

### Setting and participants

Students attending a K-12 independent urban school for students with dyslexia and related disorders, located in the southeast USA, participated in this study, with a total school enrollment of 160. Students who were invited to participate were those who were (a) of elementary age, (b) with confirmed and current diagnoses of dyslexia (or a specific learning disability in the areas of phonological processing or decoding based on psycho-educational assessments conducted by an outside evaluator required for admission to the school), (c) with normal vision, and (d) no comorbid diagnoses (e.g., attention deficit hyperactivity disorder, autism). Of the 13 students who were identified and invited, 12 returned, signed parental consent forms and participated in this study. Table 1 provides information about the age, grade, gender, ethnicity, and lexile levels of the participants. Of students that participated in the study, seven were female and five were male. Three students were in third grade, three in fourth grade, two in fifth grade, and four were in sixth grade. Their lexile levels ranged from 120 to 1137. The probes created by the authors were based on the lower lexile scores of the participants.

**Table 1:** Participants

Pseudonym	Age	Grade	Sex	Race/ethnicity	Lexile
Randy	12–4	6^th^	M	C	450–600
Steve	12–8	6^th^	M	C	250–400
Chris	12–5	6^th^	M	C	900–1050
Elizabeth	12–5	6^th^	F	C	800–950
Kelly	11–6	5^th^	F	C	500–660
Audrey	10–11	5^th^	F	C	987–1137
Michael	10–7	4^th^	M	C	500–650
Bethany	10–4	4^th^	F	C	339–489
Jake	10–2	4^th^	M	C	250–400
Ellen	9–0	3^rd^	F	C	120–350
Lilly	10–4	3^rd^	F	A	159–309
Madison	10–4	3^rd^	F	C	519–669

*M* male, *F* female, *A* Asian, *C* Caucasian

### Measures/dependent variables

Within single case research design, dependent variables should be selected for their social significance (Horner et al., 2005). Therefore, three types of reading tasks were selected because of their strong correlation to reading achievement and sensitivity to both reading accuracy and speed: (a) letter naming (Fuchs, Compton, Fuchs, Bouton, & Caffrey, 2011), (b) real word decoding, and (c) nonsense word decoding (Nunes, Bryant, & Barros, 2012; Kendeou, van den Broek, White, & Lynch, 2009). Further, these tasks directly measure the anecdotal claims made by the font developers. The letter-naming probes acted as a measure of rapid automatic naming and letter identification in isolation. The real word list provided a measure of word reading or decoding and the nonsense word list allowed for a measure of sound-symbol correspondence that was free from effects of previous reading learning and memorization. A measure of reading comprehension was not selected because reading comprehension can be influenced by many other reading and executive functioning skills unrelated to decoding of print.

#### Stimulus material

**Fig. 2:**
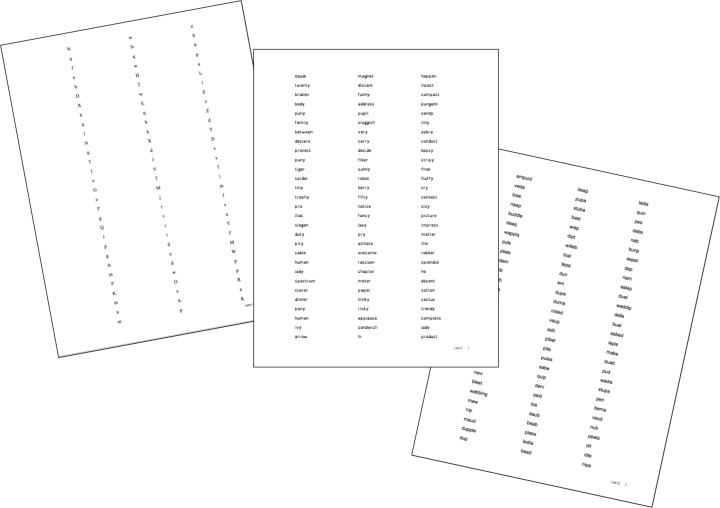
Probe examples: letter naming in TNR, words in OD, and nonsense words in Arial

Three sets of research-created probes were created, one set for each reading task: (a) letter-naming, (b) real word decoding, and (c) nonsense word decoding (See Fig. 2). The letter-naming probes contained a list of randomly ordered upper and lowercase letters. The real word list contained phonetically regular one- and two-syllable words. The nonsense word list contained nonreal words following typical orthographic patterns. From each set, seven randomly ordered lists were generated. Each list was then printed in each of the three fonts (Arial 12, OD 10, TNR 12). The nominal font sizes varied in order to keep the physical size of the font consistent across probes. Each list consisted three columns of double-spaced rows and was printed on standard white copy paper.

### Procedure

For each session, participants read a letter-naming list, a real word list, and a nonsense word list each for one minute. If a student hesitated for 4 s, he or she was prompted to skip that item and move to the next one. A digital timer with an audible alarm/bell was used to time each 1-min reading session. The researchers recorded student responses on identical observer copies of the student probes. A random-number calculator (Microsoft Excel) was used in order to randomize the font sequence to ensure the order of presentation did not affect the decoding rate or accuracy.

#### Inter-observer agreement

Each session was audio-recorded. Later, a graduate student not familiar with the research question compared the researcher-marked observer copy to the audio recording. Inter-observer agreement was calculated with the following formula: agreements/(agreements + disagreements). Inter-observer agreement was conducted on at least 96 % of all administrations with a median agreement of 100 % (range = 99.3–100 %) for letter naming, 100 % (range = of 98.2–100 %) for real word reading, and 100 % (range = of 97.9–100 %) for nonsense word reading.

### Experimental design

This study utilized an alternating treatment design (ATD), a form of single-subject research, to investigate the effects of the specialized dyslexia font, OD, compared to Arial and Times New Roman fonts on reading accuracy and speed in elementary-aged readers with dyslexia. ATD is an ideal design for comparing the effects of two or more treatments in applied research, and can identify the presence or absence of a causal relationship between the independent variable (e.g., font type) and a change in the dependent variable (e.g., reading speed and accuracy (Smith, 2012). ATDs control for internal threats to validity related to inter-subject variability by essentially dividing each participant into multiple identical participants receiving each treatment (Martella, Nelson, & Marchand-Manella, 1999). Further, ATDs that include random assignment of treatment order and replication across multiple participants rule out rival hypotheses, resulting in elegant control of internal threats to validity (Horner et al., 2005).

### Data analysis

The results of this study were analyzed through visual analysis, as well as nonparametric statistical analysis.

Visual analysis was used to evaluate the outcomes of this study. A positive effect is present when there is (a) a consistent level, trend, and variability within each phase or condition; (b) an immediate effect, proportion of overlap, consistent data across phases, and projected patterns of the dependent variable to determine the presence of an intervention effect; and (c) absence of anomalies within the data (e.g., sudden changes in level or trend).

While visual analysis has been shown to be effective in detecting large, practically important and clinically significant participant outcomes, it can be insensitive to smaller effects (Glass, 1997; Parsonson & Baer, 1992). Therefore, we also conducted statistical tests to identify and summarize the effect of each font.

Because the data did not meet the parametric assumptions (e.g., parametric assumptions of normally distributed data, homogeneity of variance of the residuals, and the independence of the distribution of the residuals; Campbell, 2004; West & Hepworth, 1991; Hersen & Barlow, 1976), a nonparametric effect size calculation was used. Distribution-free nonparametric models are not impeded by these parametric assumption violations (Parker & Vannest, 2009) and are a measure of practical significance (Parker, Vannest, & Brown, 2009).

While there are several nonparametric methods for calculating effect size for SCDs, improvement rate difference (IRD) was selected for use here because it is an intuitive approach that makes use of established effect sizes (e.g., Phi, Cohen’s Kappa and Cramer’s V). IRD is the difference in improvement rates between baseline and treatment phases (Parker et al., 2009) and is commonly used in medical research under the terms “risk difference” or “risk reduction” (Vannest, Harrison, Temple-Harvey, Ramsey, & Parker, 2010). Confidence intervals and *p* values for IRD can also be calculated using commonly available statistics modules. The strengths of IRD for single case research are described at length in Parker et al. (2009).

IRD is calculated by finding the difference between the improvement rate in baseline and the improvement rate in treatment. The improvement rate is for each phase is the number of “improved data points” divided by the total data points in that phase. In a treatment phase, an improved data point is any point that exceeds all data points in the baseline phase. Within a baseline phase, an improved data point is one that ties or exceeds any points in the treatment phase. IRD is then calculated from these two independent proportions. For this analysis we used the online calculator (Relative Risk and Risk Difference Confidence Intervals, version 1.0, Buchan, 2004; available at http://www.phsim.man.ac.uk/risk/Default.aspx).

The maximum score is 100 % IRD and occurs when there is no overlap between treatment and baseline. A 50 % IRD, or chance levels of improvement, occurs when half of the scores overlap between phases. A negative IRD score occurs when the treatment deteriorates below baseline levels (Parker et al., 2009).

Confidence intervals were also calculated around IRD to provide a measure of confidence, so that we can conclude with 95 % confidence that the true difference between the two conditions (Arial vs OD, TNR vs OD) lies within the calculated interval.

## Results

Eleven of the 12 students completed each of the three reading task probes (i.e., letter-naming, word reading, and nonsense word reading). One male student was removed from the nonsense word reading on the second day of data collection because his articulation patterns interfered with accurate scoring. His nonsense word reading results were not included in this analysis.

### Visual analysis

**Fig. 3:**
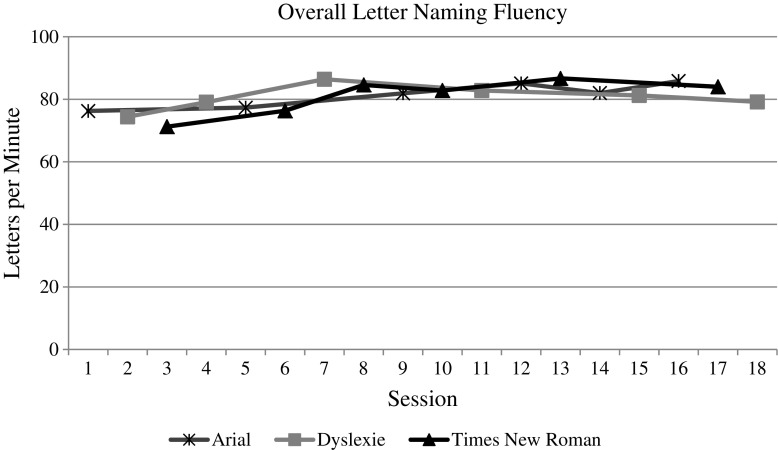
Overall average letter naming fluency (letters correct per minute). Accuracy

**Fig. 4:**
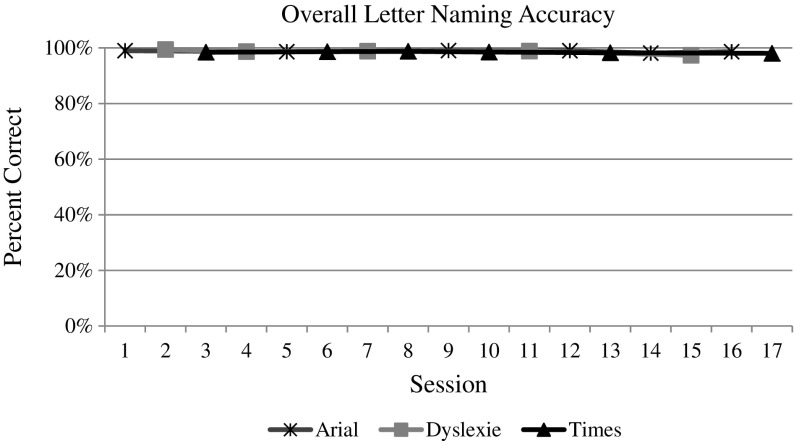
Overall average letter naming accuracy (letters correct/total attempts)

**Fig. 5:**
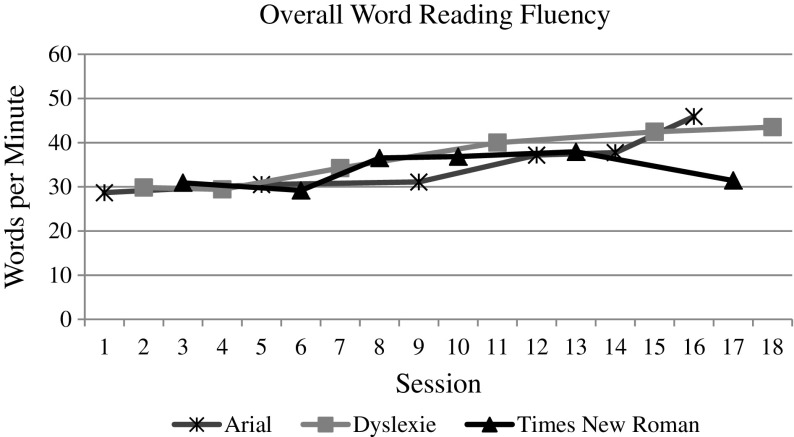
Overall average reading fluency (words correct per minute)

**Fig. 6:**
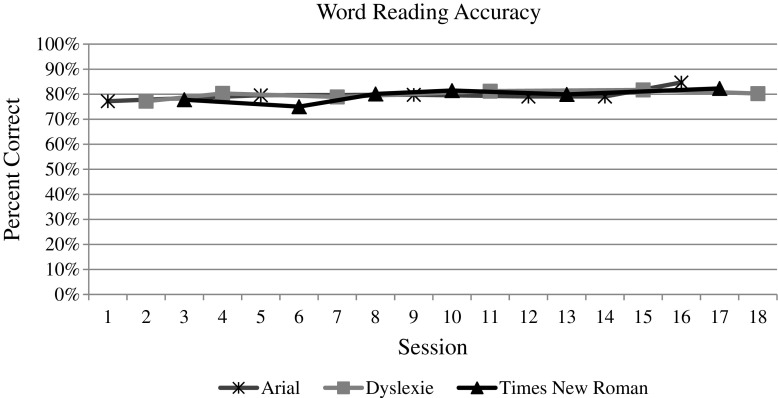
Overall average reading accuracy (letters correct/total attempts)

**Fig. 7:**
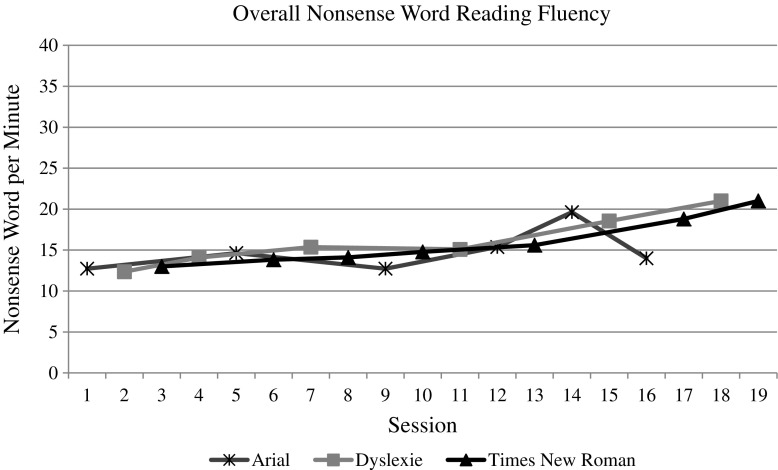
Overall average nonsense word reading fluency (pseudo-words correct per minute)

**Fig. 8:**
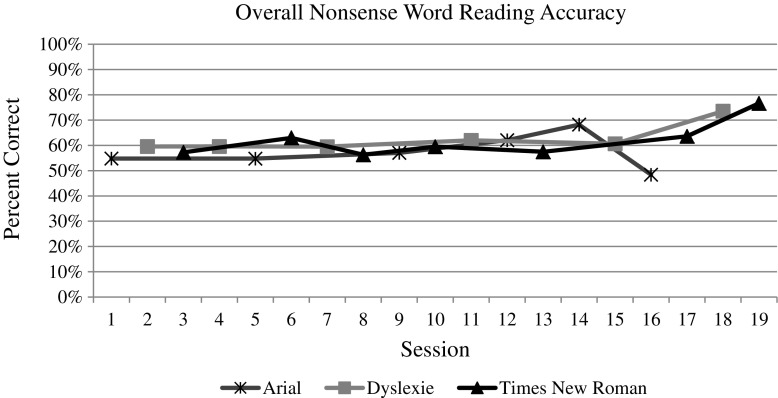
Overall average nonsense word reading accuracy (pseudo-words correct/total attempts)

The whole group data and graphs were evaluated by the visual analysis procedures described above. For both measures (i.e., speed and accuracy) of each reading task (i.e., letter naming, word reading, and nonsense word reading), the visual analysis of the data does indicate an overall increasing trend over time, as one would expect with the effect of practice (see Figs. 3, 4, 5, 6, 7, and 8). However, no individual trend line for any of the specific fonts demonstrated a stronger increasing trend than any other. Further, the significant amount of overlap between each of the individual font data lines indicates that no one font lead to significantly better or worse reading accuracy or speed. Further, individual student graphs and data were also analyzed to investigate individual effects, none of which demonstrate a positive effect (Please contact the first author for copies of individual student graphs).

### Effect size calculation

#### IRD

Arial, OD, and TNR were evaluated to determine if differences between the font in area of reading fluency and accuracy for each of the reading tasks (See Tables 2 and 3). The confidence intervals take into account the number of participants and observations, describe how reliable survey results are, and provide a range within which the parameter is likely to lie.

**Table 2:** Fluency effect (correct letters or words per minute)

Reading task	Contrast	IRD ES (%)	95 % Confidence interval
Letter naming	Arial vs OD	−49.65 %	−63.33, −32.98
	TNR vs OD	−67.73 %	−73.60, −46.17
Word reading	Arial vs OD	−88.65 %	−94.45, −77.57
	TNR vs OD	−82.81 %	−90.01, −71.74
Nonsense word reading	Arial vs OD	−69.70 %	−79.90, −55.74
	TNR vs OD	−77.24 %	−85.99, −64.10.

*OD* OpenDyslexic, *TNR* Times New Roman

**Table 3:** Accuracy effect (correct/total attempts)

Reading task	Contrast	IRD ES (%)	95 % Confidence interval
Letter naming	Arial vs OD	−68.18 %	−78.64, −54.10
	TNR vs OD	−63.62 %	−74.96, −48.66
Word reading	Arial vs OD	−53.89 %	−67.52, −36.70
	TNR vs OD	−73.53 %	−83.38, −59.28
Nonsense word reading	Arial vs OD	−67.19 %	−78.28, −52.02
	TNR vs OD	−75.81 %	−85.09, −61.90

*OD* OpenDyslexic, *TNR* Times New Roman

On measures of reading fluency, OD produced negative results, or decreased students’ outcomes compared to both Arial and TNR, on all three reading tasks (i.e., letter naming, word decoding, nonsense word decoding). ES ranged from −88.65 %, CI_95_ [−94.45, −77.57] Arial compared to OD on word reading, to −49.65 %, CI_95_ [−63.33, −32.98] Arial compared to OD on letter naming.

On measures of reading accuracy, OD also produced negative results, or decreased students’ outcomes compared to both Arial and TNR, on all three reading tasks (i.e., letter naming, word decoding, nonsense word decoding). ES ranged from −73.53 %, CI_95_ [−83.38, −59.28] TNR compared to OD on word reading, to −63.62 %, CI_95_ [−74.96, −48.66] TNR compared to OD on letter naming.

Based on visual and statistical analysis, there is “no evidence” of OD having a positive effect on reading speed or accuracy.

While not the focus of this study, it is interesting to note that there appears to be no practically significant difference between the use of TNR and Arial fonts, even though some have opined a preference for one of the other (British Dyslexia Association, n.d).

## Discussion

The development of the OD font may have been developed as the result of a common misunderstanding of dyslexia. Many new teachers believe dyslexia is caused by a deficit in visual perception (Allington, 1982; Bell, McPhillips, & Doveston, 2009; Hudson, High, & Al Otaiba, 2007; Wadlington & Wadlington, 2005; Washburn, Joshi, & Binks, 2011a, Washburn, Joshi, & Binks-Cantrell, 2011b; Washburn et al., 2013), which may have originated in a very early use of the term “word blindness” (Das & Das, 2009) and perpetuated by the fact that many students with dyslexia have letter reversals. While some with dyslexia do report difficulty with vision, there is little evidence to support that this is related to dyslexia (Christenson, Griffin, & Taylor, 2001; Fletcher, Foorman, Shaywitz, & Shaywitz, 1999). In fact, emerging readers commonly reverse letters as they consolidate and make sense of the sound-symbol system (Adams, 1998).

With the poor outcomes and personal struggles associated with dyslexia, teachers and parents are eager to find interventions to improve reading outcomes. Consequently, people in a variety of fields are seeking to help find solutions. However, to truly meet the needs of students who struggle to learn to read, they need to be provided interventions that are empirically proven to be effective. Given the press and popular support of using a specialized font as a remedy for dyslexia, it is critical to highlight that results from this study failed to identify any positive effect for using it. Currently, there is no documentation to support a specialized font is an evidence-based practice. Teachers, administrators, and parents need to be aware of the lack of empirical data supporting any positive effects of OD on reading before altering all written material into a dyslexia-friendly font. If fact, using a font with claims to improve reading for individuals with dyslexia without evidence to support this claim could result in further frustrations by teachers, parents, and individuals with dyslexia when no difference is observed after changing fonts used. Teachers and other practitioners need to be able to discriminate between those interventions that have been empirically shown to be effective from those that have not. While some may conclude that an intervention that fails to produce a positive effect may not do good, but probably does not do harm, others disagree.

Inert interventions may in fact cause other forms of harm, in depriving resources (time and financial) away from those interventions that have demonstrated efficacy. While the intervention studied here, a freely available font, does not have costs associated with purchasing it, there are financial and time cost associated with downloading it and transferring print materials to the new font. That time and resource could be use on other interventions that are more likely to improve students’ reading ability. Further, the use of unsubstantiated interventions can impact the credibility of the profession, and lead to the public losing trust in special educators (Lilienfeld, Lynn, & Lohr, 2015).

Finally, the most harm may come when students who have already experienced significant struggle and academic failures related to learning to read, have yet another experience with failure when they are not able to read significantly better in a font designed to do so. A repeated failure experience can further damage students’ self-efficacy and academic self-esteem. Instead, students with dyslexia need well-qualified teachers and interventionists, who can skillfully implement intensive instruction (Moats, 2009). This intensive systematic intervention is likely to include direct multisensory instruction in the areas of phonological awareness, phonics, and fluency (Mather & Wendling, 2012; Shaywitz, 2003; Snowling & Hulme, 2012) that is both sequential and cumulative, and taught to automaticity (Moats, 2009).

## Limitations and future research

While all three reading tasks were selected because of their sensitivity to small changes, and nonsense word reading was selected because it reduces the influence of memorization, we did not measure comprehension of connected text—the end goal of reading. However, this study failed to find any positive effect of the specialized dyslexia font on the reading accuracy and speed, we can assume it will also have no effect on reading comprehension, this study did not directly measure that variable.

While single case research designs are empirical research designs, multiple independent studies with similar results are needed to deem any intervention or practice to be “evidence-based” (Kratochwill et al., 2010) Future research is needed to verify the results of this study.

While OD has not been shown to be an effective intervention for students with dyslexia, that is not to say the field should not continue to search for and develop new and innovative ways to improve outcomes for students. Since students with dyslexia are already behind their nondisabled peers in regards to reading achievement, it become more critical that educators use interventions empirically proven to be effective. Educators cannot waste time with interventions having no empirical support. Our children do not need to experience another failure.
